# Hydroxy-functionalized hyper-cross-linked ultra-microporous organic polymers for selective CO_2_ capture at room temperature

**DOI:** 10.3762/bjoc.12.185

**Published:** 2016-09-02

**Authors:** Partha Samanta, Priyanshu Chandra, Sujit K Ghosh

**Affiliations:** 1 Indian Institute of Science Education and Research (IISER), Pune. Dr. Homi Bhabha Road, Pashan, Pune-411008, India. Fax: +91 20 2589 8022; Tel: +91 20 2590 8076; 2 Centre for Research in Energy & Sustainable Materials, IISER Pune, Pashan, Pune, India

**Keywords:** carbon dioxide capture, hyper-cross-linked polymer, metal-organic framework, microporous organic polymer

## Abstract

Two hydroxy-functionalized hyper-cross-linked ultra-microporous compounds have been synthesized by Friedel–Crafts alkylation reaction and characterised with different spectroscopic techniques. Both compounds exhibit an efficient carbon dioxide uptake over other gases like N_2_, H_2_ and O_2_ at room temperature. A high isosteric heat of adsorption (*Q*_st_) has been obtained for both materials because of strong interactions between polar –OH groups and CO_2_ molecules.

## Introduction

The increase in the earth’s average temperature, also termed as global warming, is mainly due to the effects of greenhouse gases. The impacts of global warming includes rising sea level, more likelihood of extreme events (like floods, hurricanes etc.), widespread vanishing of animal population, loss of plankton due to warming seas. There are many heat-trapping greenhouse gases present in the atmosphere (from methane to water vapour), but CO_2_ puts us at the greatest risk if it continues to accumulate in the atmosphere. This is due to the fact that CO_2_ remains in the atmosphere in a time scale of hundred years in contrast to other greenhouse gases which leave the atmosphere with relatively smaller time scale [1]. The CO_2_ long life in the atmosphere provides the clearest possible rationale for carbon dioxide capture and storage. Previously, different types of amine solvents were employed to study the CO_2_ capture, but the need of high energy to regenerate the amine solutions after CO_2_ capture, hinders their applications further [2]. In the domain of porous materials, zeolites, metal-organic frameworks (MOFs), cage molecules, etc. have been introduced for selective uptake of CO_2_ [3–5]. In terms of surface area, tuneable porosity and feasible host–guest interaction, MOFs have scored over other above mentioned porous materials [6]. But the less hydrolytic stability of metal-organic frameworks limits their real time application [7–8]. So the search for new materials having high surface area and feasible interaction with carbon dioxide like MOFs and with high chemical stability have become one of top priority for researchers.

**Scheme 1:**
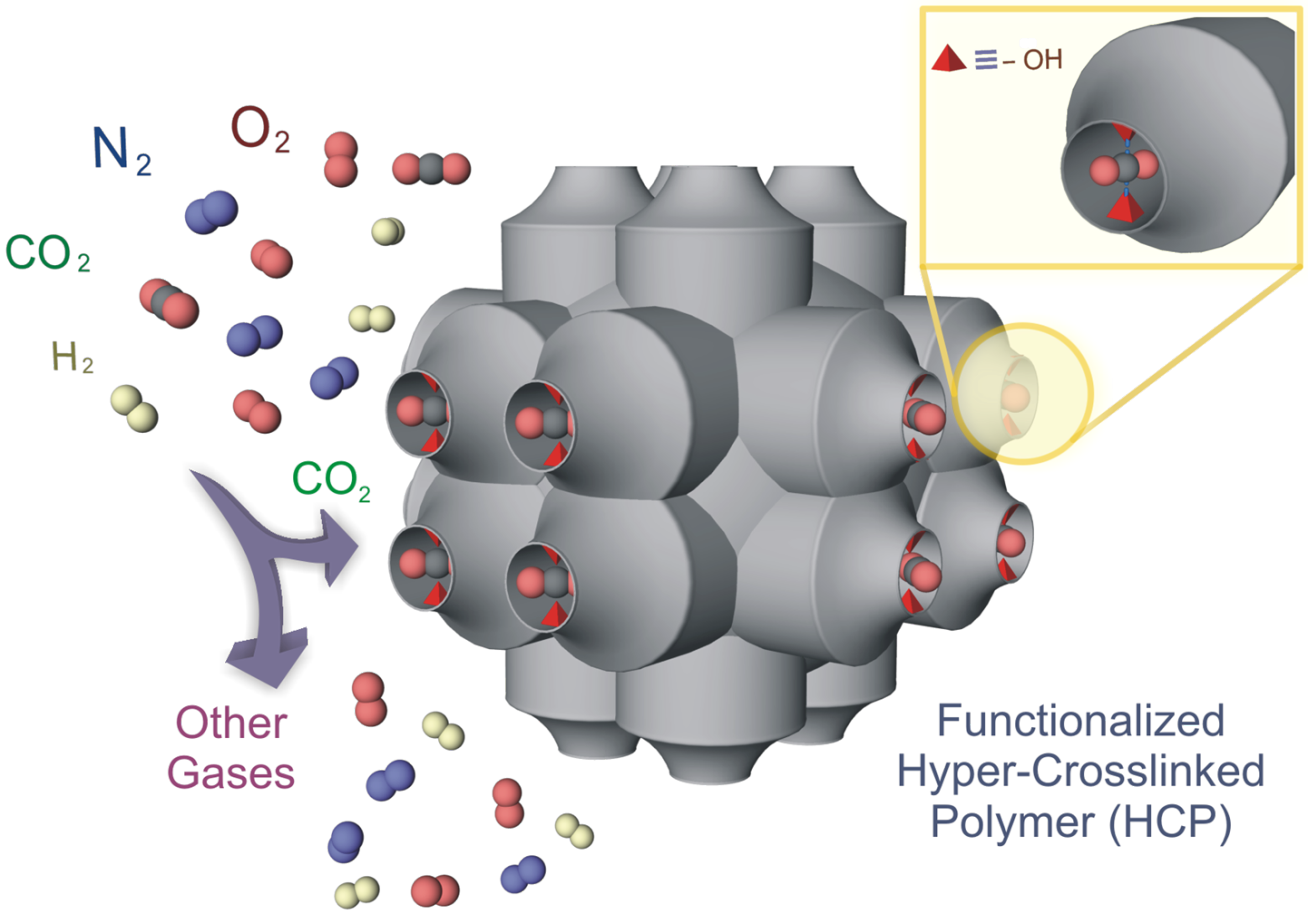
Schematic representation of selective CO_2_ capture in a porous material.

Microporous organic polymers (MOP) are a relatively new class of porous materials, constructed from light elements like H, C, B, N, O etc. having a large surface area, small pore size and low skeletal density [9–12]. This type of materials has already been used for various purposes of applications such as gas storage, gas separation, catalysis, sensing, clean energy, etc. [13–18]. Relatively weaker coordination bonds in MOFs have been replaced with stronger covalent bonds in this type of porous compounds. This results in a high chemical stability of the microporous organic polymers, which is an essential condition for the real-time application of any compound. The last decade has witnessed advancements in synthesizing various types of microporous organic materials including covalent organic frameworks (COFs), conjugate microporous polymers (CMPs), porous polymeric networks (PPNs), porous aromatic frameworks (PAFs), covalent triazine framework (CTFs), etc. [19–24]. Hyper-cross-linked microporous organic polymers (HCPs) are a subclass of this type of porous materials. Recently, hyper-cross-linked MOPs are emerged as a new subclass, synthesized by hyper-cross linking of basic small organic building blocks by Friedel–Crafts reaction in the presence of the Lewis acid FeCl_3_ (as catalyst) and formaldehyde dimethyl acetal (FDA) as the cross linker [25–27]. Here, aromatic small organic compounds are used to polymerise via C–C cross coupling to produce the targeted porous and physicochemical stable organic hyper-cross-linked polymeric materials. One huge advantage of this material is the low-cost synthesis, the cost-effective formaldehyde dimethyl acetal (FDA), FeCl_3_ and that organic small molecules can produce very low cost materials with high yield [28]. Hyper-cross-linking prevents the close packing of polymeric chains in this type of material to impart the intrinsic porosity. Hyper-cross-linked polymers have been applied in the field of gas storage, catalysis, separation and recently also in CO_2_ capture [29–32]. The increasing environmental pollution due to carbon dioxide, urges us to develop new materials with high stability, which are cost-effective and demonstrate a high efficiency in CO_2_ capture. Based on the interaction of Lewis basic sites with carbon dioxide it has been observed that porous materials functionalised with –NH_2_ groups or –OH groups exhibit a selective uptake of CO_2_ in contrast to other gases [33–34] (Scheme 1). Inspired by this we have designed and synthesized two hydroxy-functionalised hyper-cross-linked microporous organic polymers for selective CO_2_ capture at room temperature. Both compounds (HCP-91 and HCP-94) were synthesized via hyper-cross-linked C–C coupling of hydroxyl-functionalised aromatic rings by using a Friedel–Craftys reaction. At different temperatures (273 K and 298 K) gas (CO_2_, N_2_, H_2_ and O_2_) adsorption experiments were carried out for both compounds. HCP-91 and HCP-94 showed selective CO_2_ capture at both temperatures over other flue gases.

## Results and Discussion

**Figure 1:**
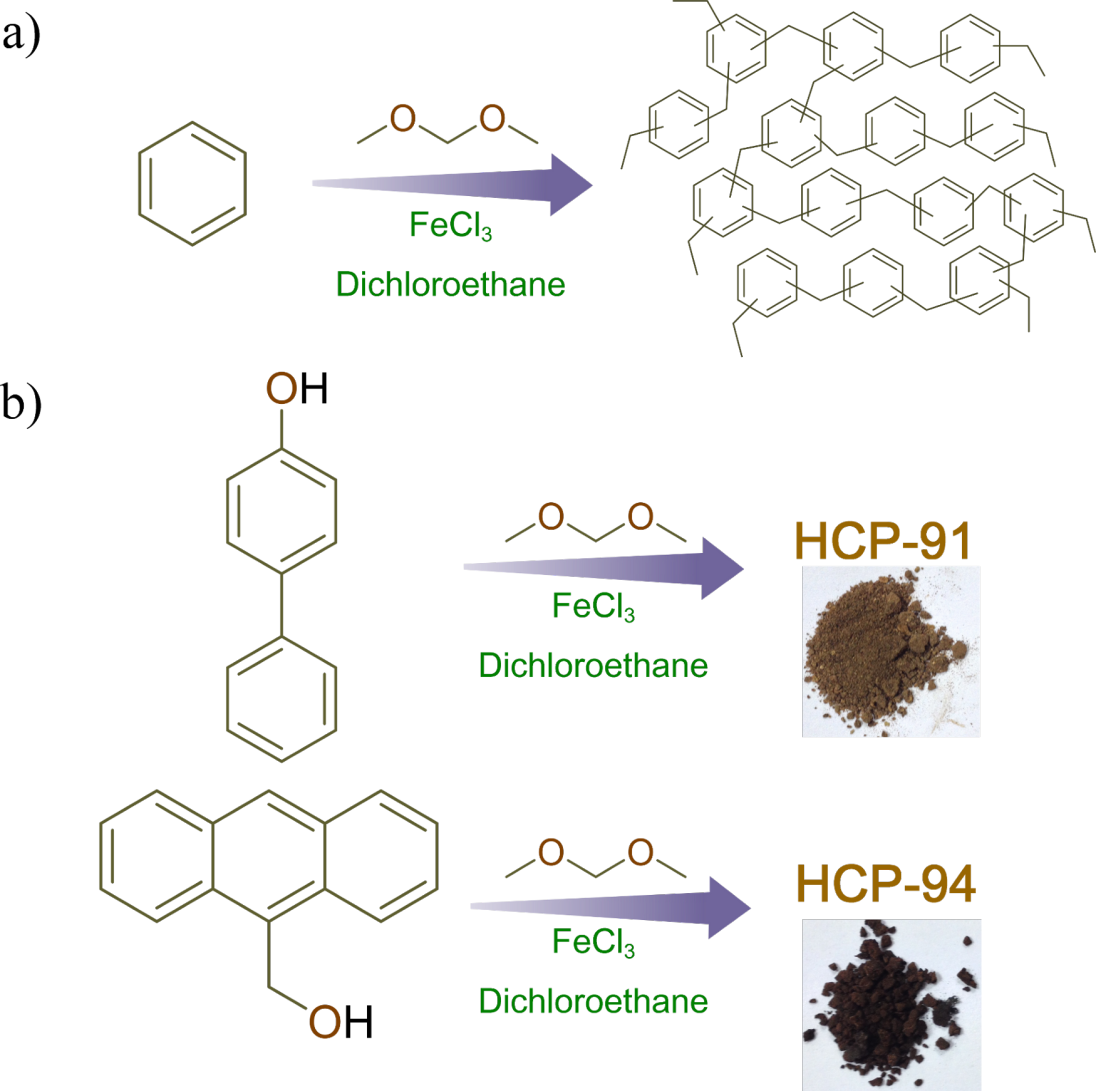
a) General synthesis scheme for hyper-cross-linked polymers (HCPs) and b) synthesis schemes for HCP-91 and HCP-94.

**Figure 2:**
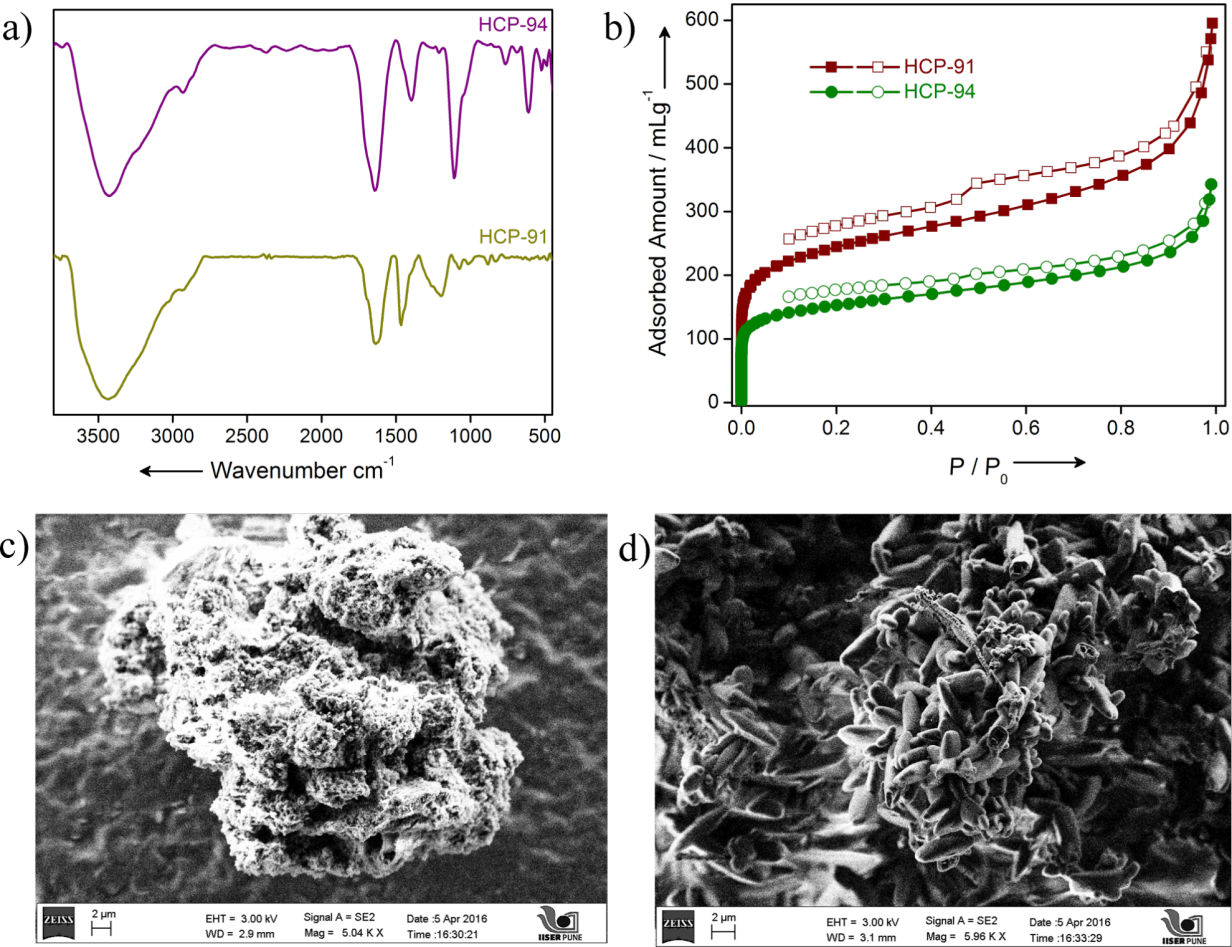
a) Infra-red spectra of HCP-91 (dark yellow) and HCP-94 (purple); b) N_2_ adsorption isotherms for HCP-91 (wine red) and HCP-94 (green) at 77 K; c) SEM image of HCP-91 and d) SEM image of HCP-94.

For the synthesis of HCP-91 and HCP-94, we used 4-phenylphenol and 9-(hydroxymethyl)anthracene, respectively (Figure 1). HCP-91 and HCP-94 have been synthesized by using a Friedel–Crafts alkylation reaction. The thus obtained as-synthesized compounds were washed repeatedly with dimethylformamide (DMF), methanol, water, chloroform, dichloromethane and tetrahydrofuran (THF) to obtain phase-pure hyper-cross-linked polymers. Both compounds were immersed in a CHCl_3_–THF (1:1) mixture and kept for 4–5 days to exchange the high boiling solvents occluded inside the framework with low boiling CHCl_3_ and THF. The solvent-exchanged phases of HCP-91 and HCP-94 were then heated at ≈100 °C under vacuum to get the guest-free desolvated phases of the respective compounds. Infrared (IR) spectroscopy was done first to characterize the constituents of both compounds. A broad peak at ≈3000–3500 cm^−1^ and two sharp peaks at ≈1465 and ≈1527 cm^−1^ can be observed in HCP-91 corresponding to the stretching frequencies of –OH groups and aromatic C=C double bonds, respectively (Figure 2a). Similar to the HCP-91, peaks corresponding to –OH groups and aromatic C=C double bonds were found at ≈3300–3500 cm^−1^ and ≈1643 and 1500 cm^−1^, respectively (Figure 2a). Meanwhile a thermogravimetric analysis (TGA) was performed with both as-synthesized and desolvated phases for HCP-91 and HCP-94. Because of the occluded solvents in the as-synthesized phases of HCP-91 and HCP-94, an initial weight loss of ≈8% and ≈10% was observed in the TGA, respectively (Figures S1 and S2 in Supporting Information File 1). Upon desolvation guest-free phases were obtained and in the TG curve a negligible loss was obtained up to ≈350 °C and ≈250 °C for HCP-91 and HCP-94, respectively (Figures S1 and S2). As confirmation of the local structures of the compounds, we performed solid state ^13^C NMR measurements (Figures S3 and S4). To investigate the morphology of both compounds we performed a field emission scanning electron microscope (FESEM) study. The morphology of HCP-91 can be described as agglomerated particles consisting of small particles without any distinct shape (Figure 2c and Figure S5). But in case of HCP-94, a clear capsule-type of morphology was found in the FESEM (Figure 2d and Figure S6).

**Figure 3:**
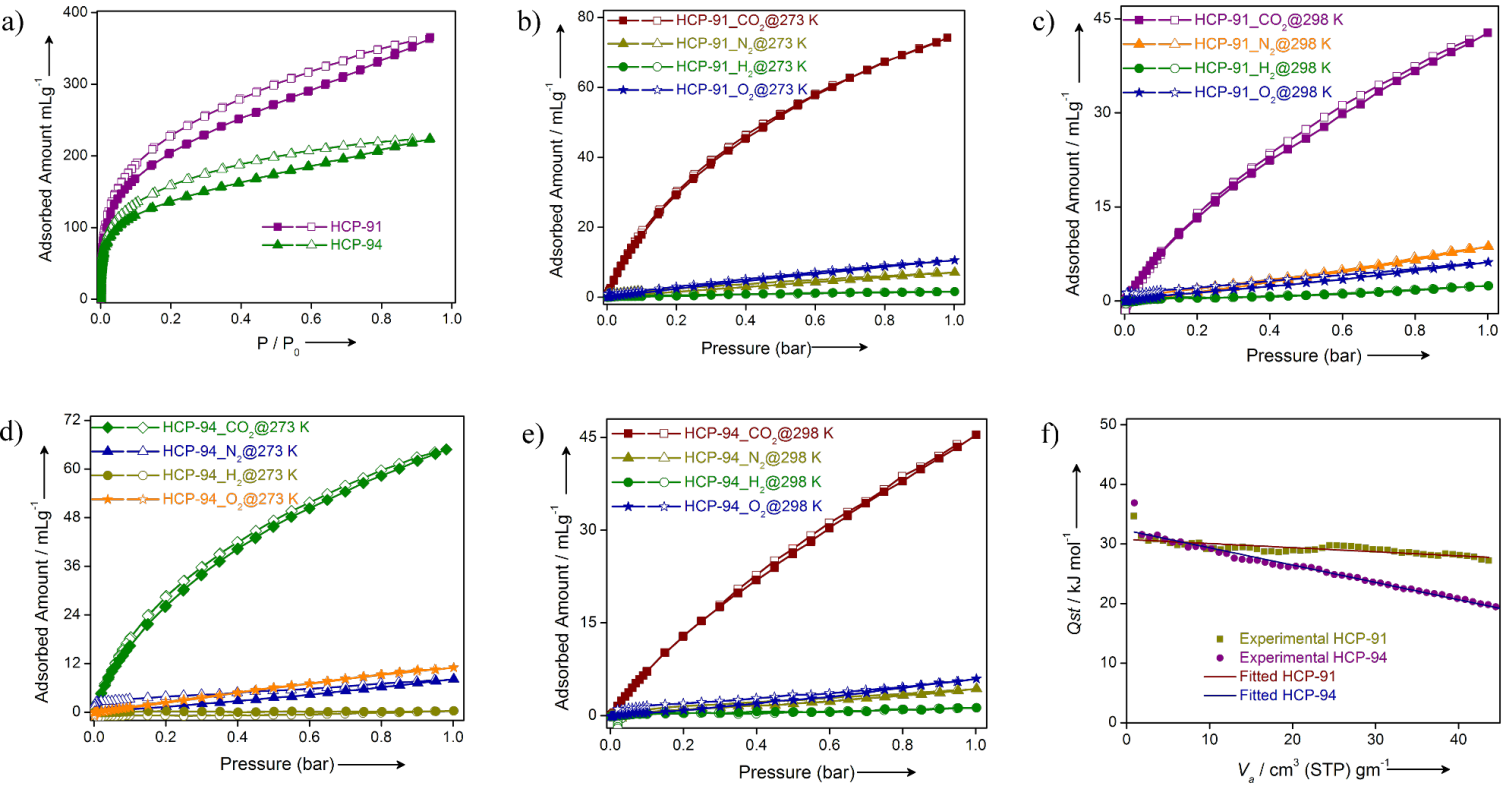
a) CO_2_ adsorption isotherms for HCP-91 (purple) and HCP-94 (green) at 195 K; b) adsorption isotherms of CO_2_ (wine red), N_2_ (dark yellow), H_2_ (green) and O_2_ (blue) for HCP-91 at 273 K; c) adsorption isotherms of CO_2_ (purple), N_2_ (orange), H_2_ (green) and O_2_ (blue) for HCP-91 at 298 K; d) adsorption isotherms of CO_2_ (green), N_2_ (blue), H_2_ (dark yellow) and O_2_ (orange) for HCP-94 at 273 K; e) adsorption isotherms of CO_2_ (wine red), N_2_ (dark yellow), H_2_ (green) and O_2_ (blue) for HCP-94 at 298 K and f) *Q_st_* plots for HCP-91 (dark yellow) and HCP-94 (purple).

After all characterizations and proper desolvation of both compounds, we investigated their porosity. First, we measured the N_2_ adsorption at 77 K. The N_2_ uptake for HCP-91 was found to be 595 mL/g, whereas that for HCP-94 was 342 mL/g (Figure 2b). Both low temperature N_2_ adsorption isotherms were of type-I category and a hysteresis was observed in desorption profiles. The hysteresis in the desorption curves can be explained in terms of a network swelling in the presence of condensed nitrogen [34]. The Howarth–Kawazoe pore-size distributions were calculated from low-temperature N_2_ adsorption data. HCP-91 and HCP-94 exhibit pore sizes of 0.59 and 0.46 nm, respectively (Supporting Information File 1, Figures S7 and S8). According to recent literature, both compounds belong to the ultra-microporous material domain as pore sizes for the above mentioned compounds are lesser than 0.7 nm [35]. Carbon dioxide uptakes of 365 mL/g and 224 mL/g for HCP-91 and HCP-94, respectively, were observed when the CO_2_ adsorption was carried out at 195 K (Figure 3a). The hysteresis in the CO_2_ desorption profile in case of both compounds accounts for the interaction between hydroxy groups and CO_2_ molecules [33–34]. Since both compounds are ultra-microporous in nature, BET (Brunauer–Emmett–Teller) surface areas were calculated from the CO_2_ adsorption profile at 195 K (Supporting Information File 1, Table S1).

The effective CO_2_ uptake at 195 K encouraged us to perform a CO_2_ adsorption study at room temperature. HCP-91 and HCP-94 both exhibit an adequate amount of carbon dioxide uptake at 273 K and 298 K (Figures S9 and S10, Supporting Information File 1). At 273 K the CO_2_ uptake was 74 mL/g for HCP-91 and 65 mL/g for HCP-94 at 1 bar (Figure 3b,d). In the case of CO_2_ adsorption at 298 K a similar uptake has been observed for both compounds: 43 mL/g (HCP-91) and 45 mL/g (HCP-94) at 1 bar (Figure 3c,e). The uptake amounts of CO_2_ at room temperature and 1 bar are comparable with other well performing microporous polymer compounds. Meanwhile adsorption of other gases like nitrogen, hydrogen and oxygen (constituents of air) were performed at 273 K and 298 K and 1 bar. Interestingly very negligible amounts of uptake were obtained for each of them (Figure 3b–e). This type of CO_2_ separation over other flue gases at room temperature can be attributed to the high interaction of carbon dioxide with the framework. Both hyper-cross-linked polymers have hydroxy groups which are polar in nature. On the other hand CO_2_ molecules have a quadrupole moment, which renders a positive charge density over the carbon atom. So the polar hydroxy groups can offer a strong dipole-quadrupole moment interaction with carbon dioxide molecules. For a better understanding of the interaction between CO_2_ and our HCPs materials, we calculated the isosteric heat of adsorption (*Q_st_*) for CO_2_. Heat of adsorptions for both compounds has been calculated from CO_2_ adsorption data at 273 K and 298 K by using the Clausius–Clapeyron equation (Figure 3f) [33]. The *Q_st_* values for HCP-91 and HCP-94 were found to be 30.7 kJ mol^−1^ and 32 kJ mol^−1^, respectively. According to the previous reports, this high isosteric heat of adsorption values for both the materials indicates the strong interaction of it with CO_2_ molecules.

## Conclusion

In this report, we have synthesized two hyper-cross-linked ultra-microporous organic polymers (HCP-91 and HCP-94) by following a cost-effective and easy synthesis route. One step Friedel–Crafts syntheses were carried out by using hydroxy-functionalized organic building blocks. Both compounds were characterised thoroughly by IR spectroscopy, TG analysis, solid state ^13^C NMR technique, FESEM and adsorption measurements. An efficient selective carbon dioxide capture was obtained for both compounds over other flue gases. High *Q_st_* values for both compounds ascribed the strong dipole–quadrupole interaction between polar –OH groups and CO_2_ molecules. We believe that this result will stimulate further design and fabrication of such low cost materials to be used as carbon dioxide capture materials.

## Supporting Information

The Supporting Information contains the experimental section, thermo-gravimetric analysis curves, solid state ^13^C NMR, FESEM images, pore size distribution plots and room temperature CO_2_ adsorption plots.

File 1 Experimental and analytical data.
