# Supplementary material for: Hydroxy-functionalized hyper-cross-linked ultra-microporous organic polymers for selective CO2 capture at room temperature
Source: Beilstein J Org Chem. 2016 Sep 2;12:1981–6. doi: 10.3762/bjoc.12.185 (PMC5082474; doi:10.3762/bjoc.12.185)
Supplement: File 1 — Experimental and analytical data. [file Beilstein_J_Org_Chem-12-1981-s001.pdf]

Supporting Information

for

Hydroxy-functionalized hyper-cross-linked ultra-microporous organic polymers for selective CO_2 capture at room temperature

Partha Samanta[1], Priyanshu Chandra[1] and Sujit K. Ghosh*[1,2]

Address: [1]Indian Institute of Science Education and Research (IISER), Pune. Dr. Homi Bhabha Road, Pashan, Pune-411008, India. Fax: +91 20 2589 8022; Tel: +91 20 2590 8076 and [2]Centre for Research in Energy & Sustainable Materials, IISER Pune.

Email: Sujit K. Ghosh*- sghosh@iiserpune.ac.in
*Corresponding author

Experimental and analytical data

Table of Contents

Experimental section **S2**

Figures S1 and S2: TGA data **S4**

Figures S3 and S4: Solid state ^{13}C NMR **S6**

Figures S5 and S6: FESEM images **S8**

Figures S7 and S8: HK pore size distribution **S10**

Figures S9 and S10: Gas adsorption data **S12**

Table S1 **S14**

Experimental section:

Materials: 4-Phenylphenol, 9-(hydroxymethyl)anthracene, $FeCl_3$ and formaldehyde dimethyl acetal were purchased from Sigma-Aldrich. Solvents were obtained locally. These chemicals were used without further purification.

Physical measurements: The IR spectra were acquired on a NICOLET 6700 FTIR spectrophotometer using KBr pellets in a 400–4000 cm^{-1} range. Thermogravimetric analyses (TGA) were recorded on a Perkin-Elmer STA 6000, TGA analyser under N_2 atmosphere with a heating rate of 10 °C/min. Gas adsorption measurements were studied using a BelSorp-max instrument from Bel Japan. FESEM was done by using aFEI Quanta 3D dual beam ESEM at 30KV.

Synthesis of HCP-91:

HCP-91 has been synthesized by cross-coupling of 4-phenylphenol (Figure 1). In a round bottom flask 4-phenylphenol (300 mg, 1.7625 mmol) was placed and to that 20 mL of dichloroethane ($C_2H_4Cl_2$) was added. Then to the reaction mixture formaldehyde dimethyl acetal (470 µL, 5.288 mmol) and $FeCl_3$ (860 mg, 5.288 mmol) were added, respectively. The reaction mixture was heated at 50 °C for 5 hours and then was allowed to reflux at 80 °C for 20 hours (Scheme S1). On completion of the reaction brown coloured precipitate was filtered off and washed with DMF, methanol, water, chloroform, dichloromethane and tetrahydrofuran (THF) repeatedly. Thus obtained brown coloured solid material was then kept in a 1:1 $CHCl_3$–THF mixture (25 mL) for 3 days to remove the high boiling solvents from the porous network of HCP-91. Then the solvent exchanged phase of HCP-91 was heated at 100 °C under vacuum to obtain the solvent-free activated material and with this phase further works have been carried out. Yield: 365 mg.

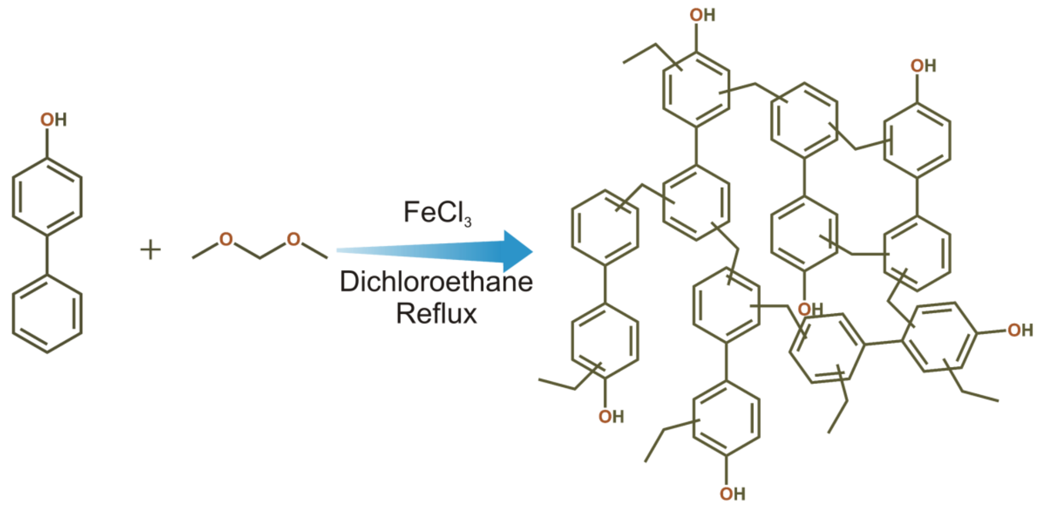

Scheme S1: Synthesis of HCP-91.

<u>Synthesis of HCP-94:</u>

In a round bottom flask 9-(hydroxymethyl)anthracene (250 mg, 1.2 mmol) was dissolved in 25 mL of dichloroethane. After that formaldehyde dimethyl acetal (477 μL, 5.4 mmol) and $FeCl_3$ (877 mg, 5.4 mmol) were added respectively to the reaction mixture. The reaction mixture was then allowed to heat at 50 °C and then refluxed at 80 °C for 24 hours (Scheme S2). After 24 hours reaction was cooled to room temperature and black precipitate was filtered off. Thus obtained black colored powder was washed with DMF, THF, water, $CHCl_3$ and methanol. Later to exchange the high boiling solvents inside the framework, it has been kept in 1:1 $CHCl_3$–THF mixture for 3 days and then solvent exchanged phase was heated at 100 °C under vacuum to get the activated phase of HCP-94. Yield: 325 mg.

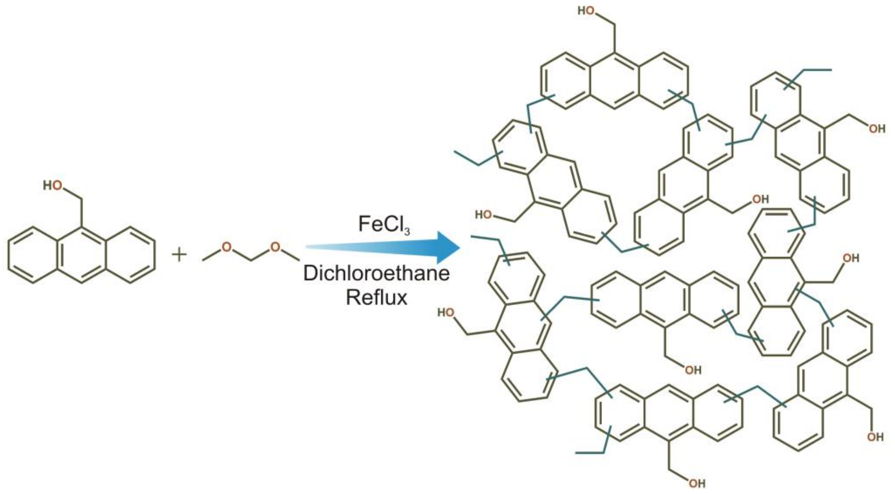

Scheme S2: Synthesis of HCP-94.

Figures S1 and S2: TGA data

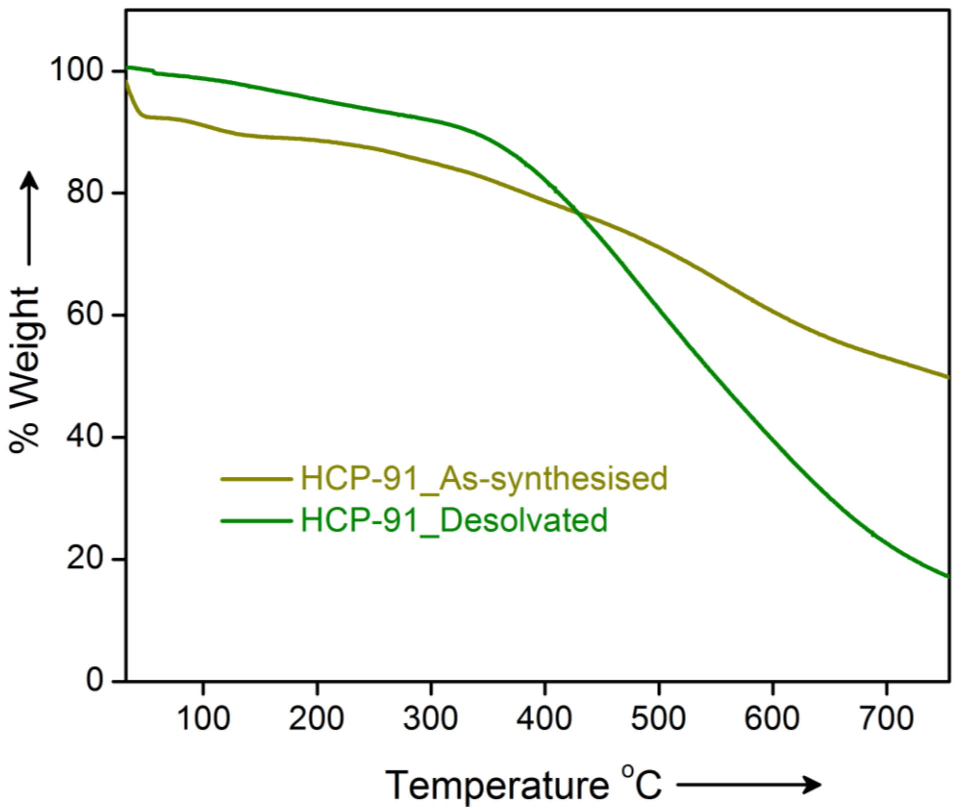

Figure S1: Thermo-ravimetric analysis of as-synthesised phase (dark yellow) and desolvated phase (green) of HCP-91.

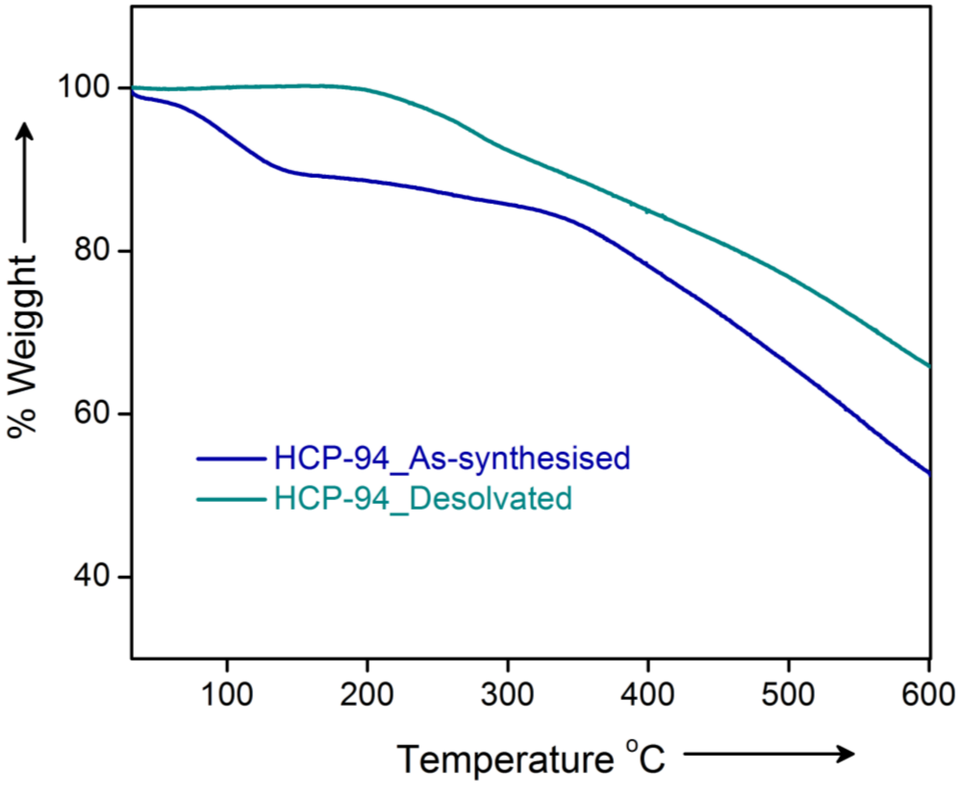

Figure S2: Thermogravimetric analysis of as-synthesised phase (blue) and desolvated phase (cyan) of HCP-94.

Figures S3 and S4: Solid state ^{13}C NMR

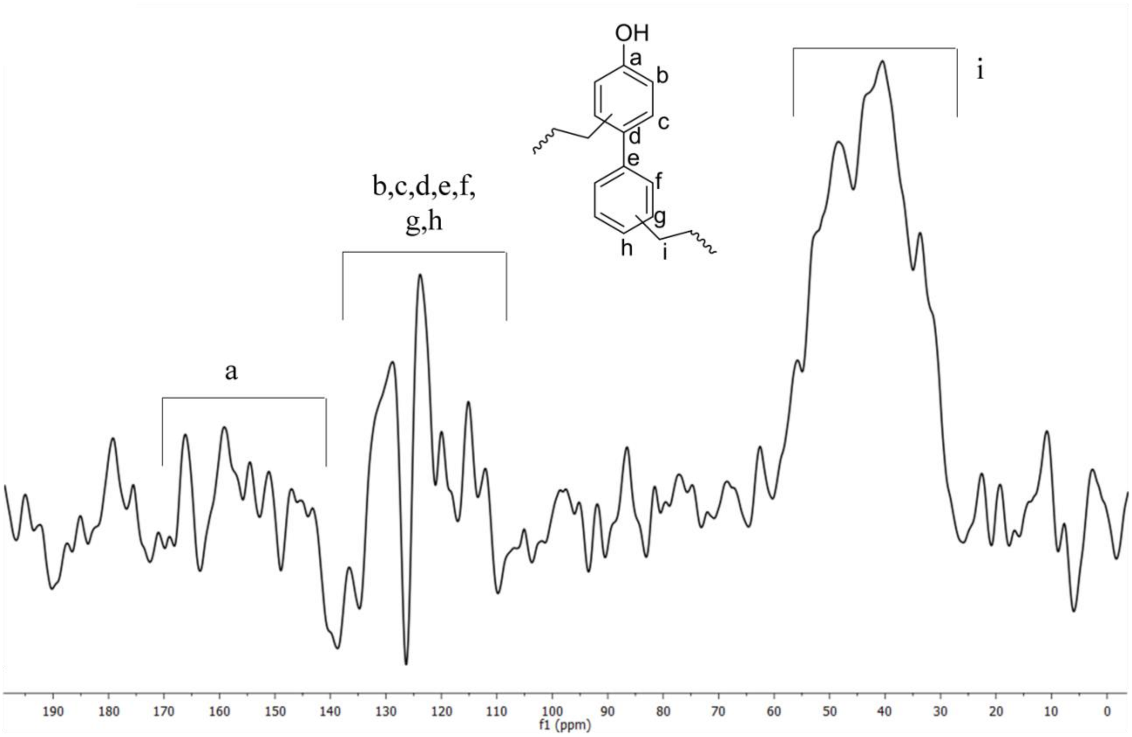

Figure S3: Solid-state ^{13}C NMR of HCP-91.

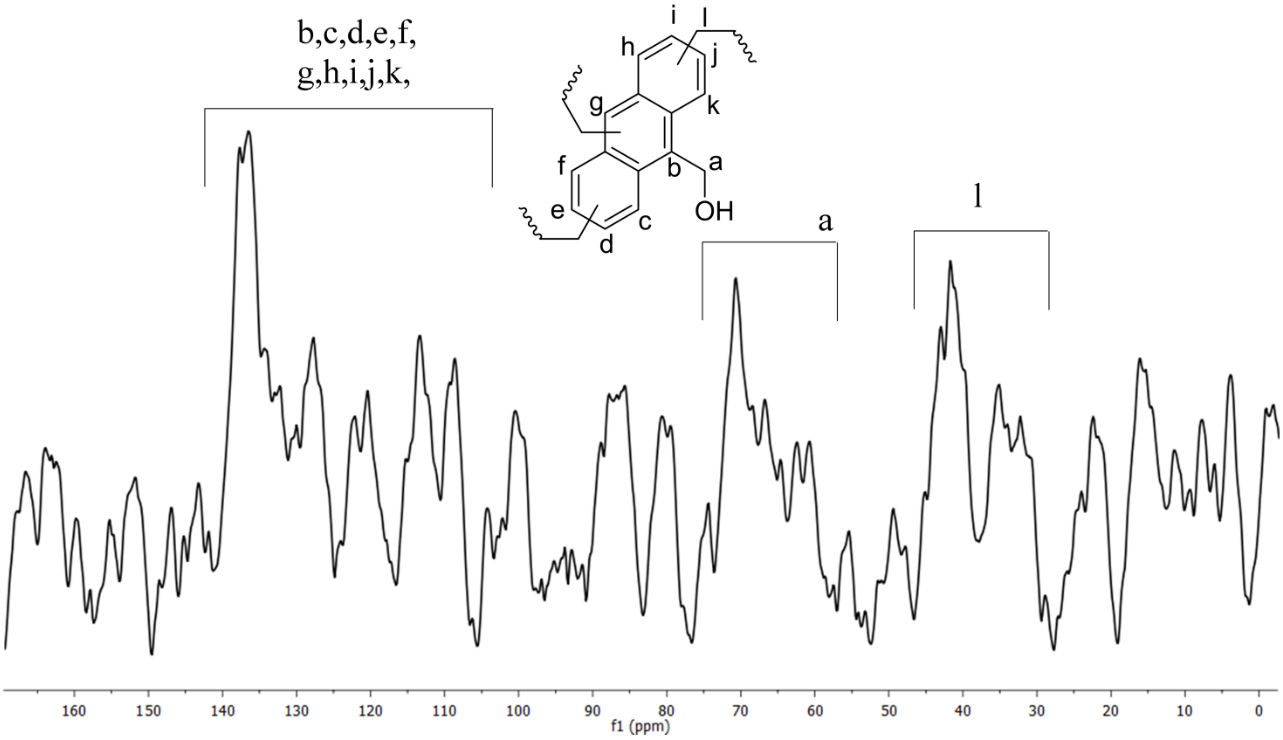

Figure S4: Solid-state ^{13}C NMR of HCP-94.

Figures S5 and S6: FESEM images

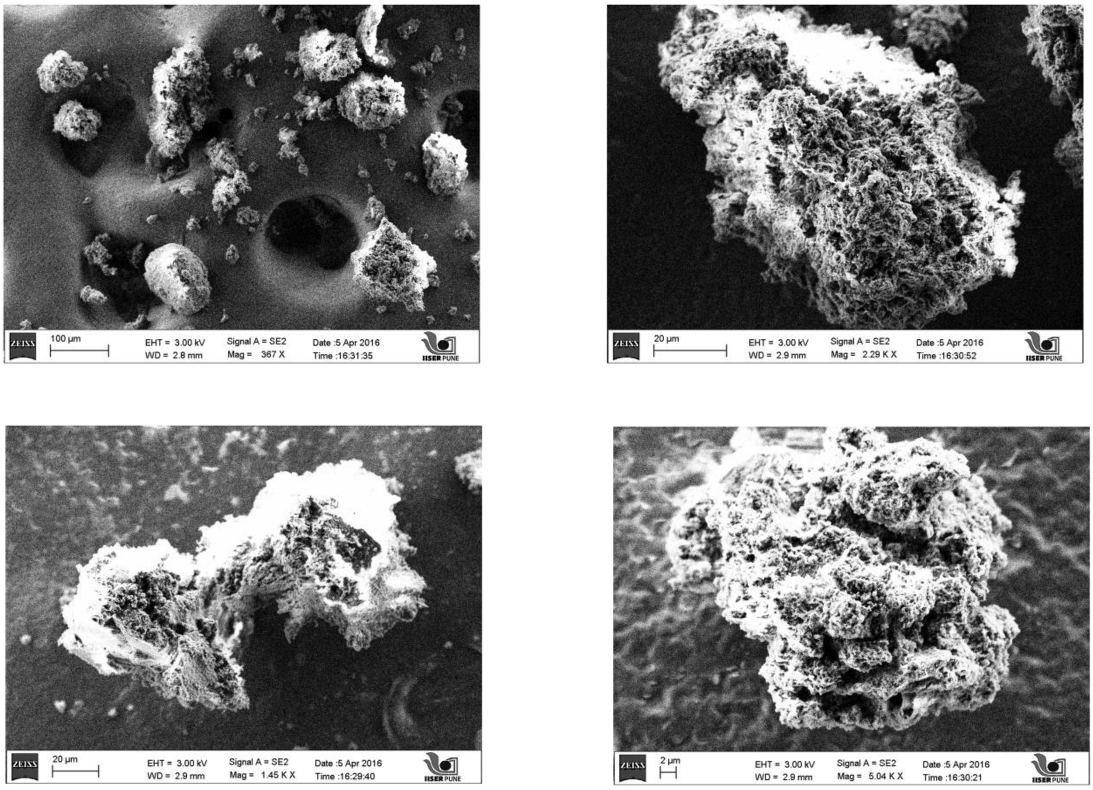

Figure S5: FESEM images of HCP-91.

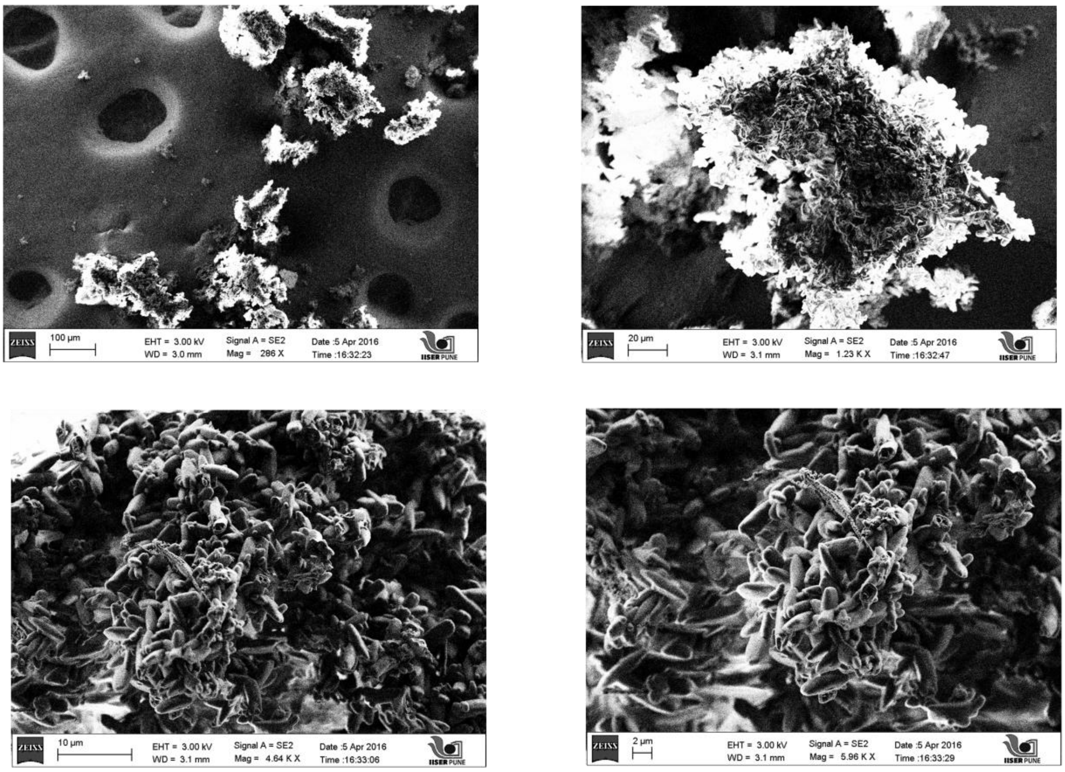

Figure S6: FESEM images of HCP-94.

Figures S7 and S8: HK pore size distribution

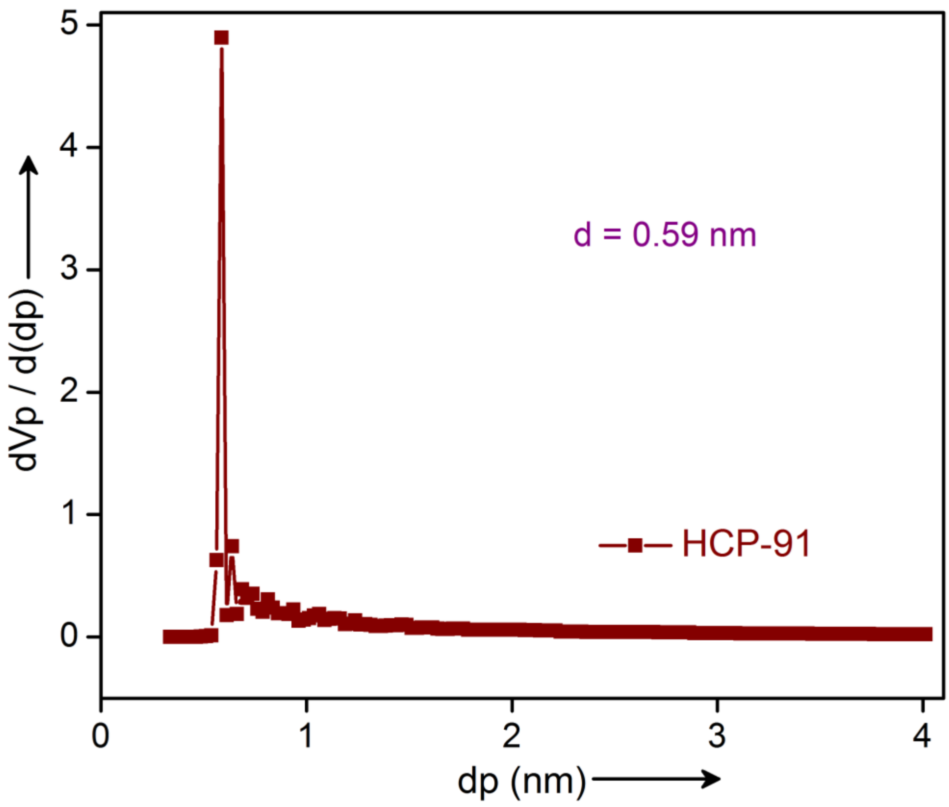

Figure S7: Howarth–Kawazoe (HK) pore-size distribution calculated from N_2 adsorption at 77 K for HCP-91.

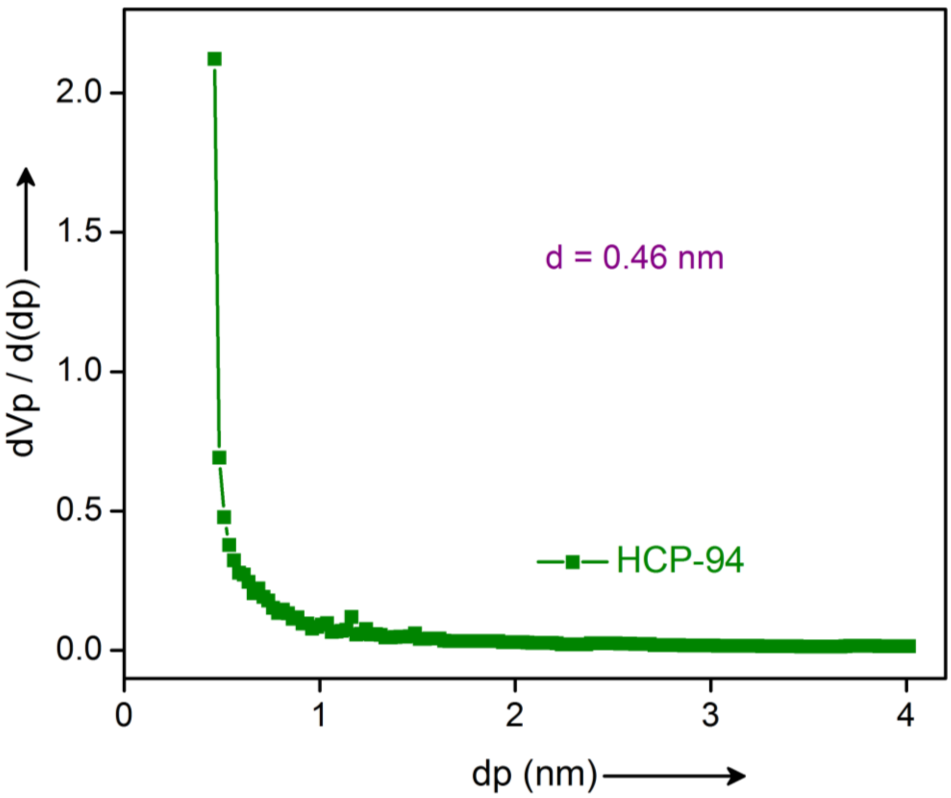

Figure S8: Howarth–Kawazoe (HK) pore-size distribution calculated from N_2 adsorption at 77 K for HCP-94.

Figures S9 and S10: Gas adsorption data

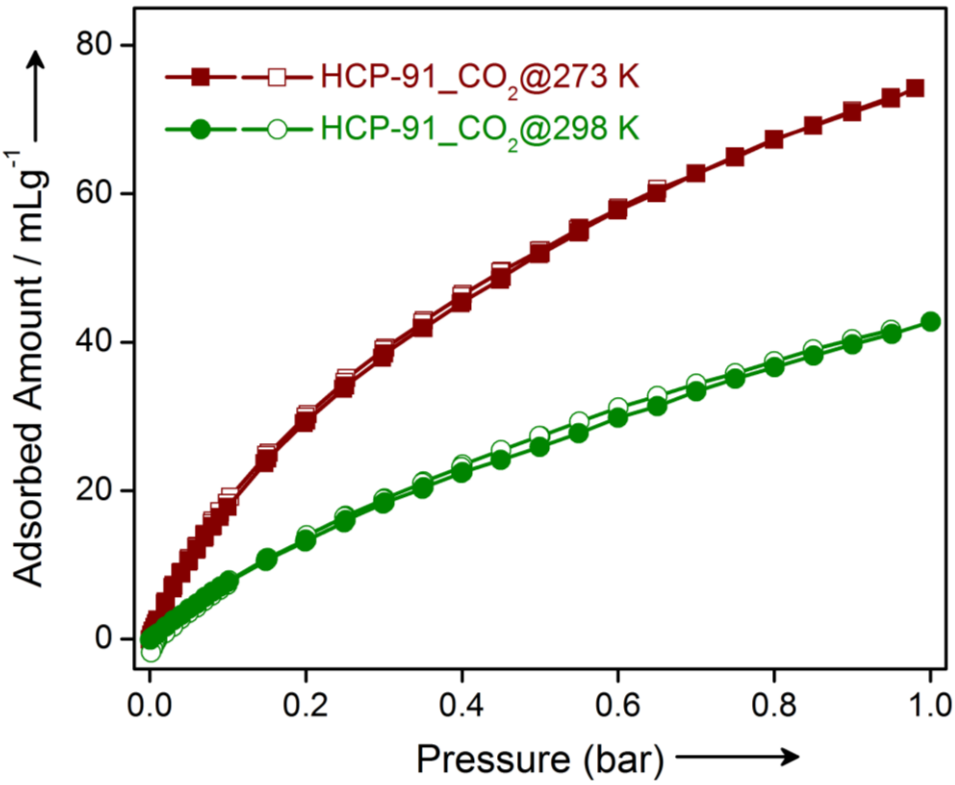

Figure S9: CO_2 adsorption isotherms at 273 K (wine red) and 298 K (green) for HCP-91.

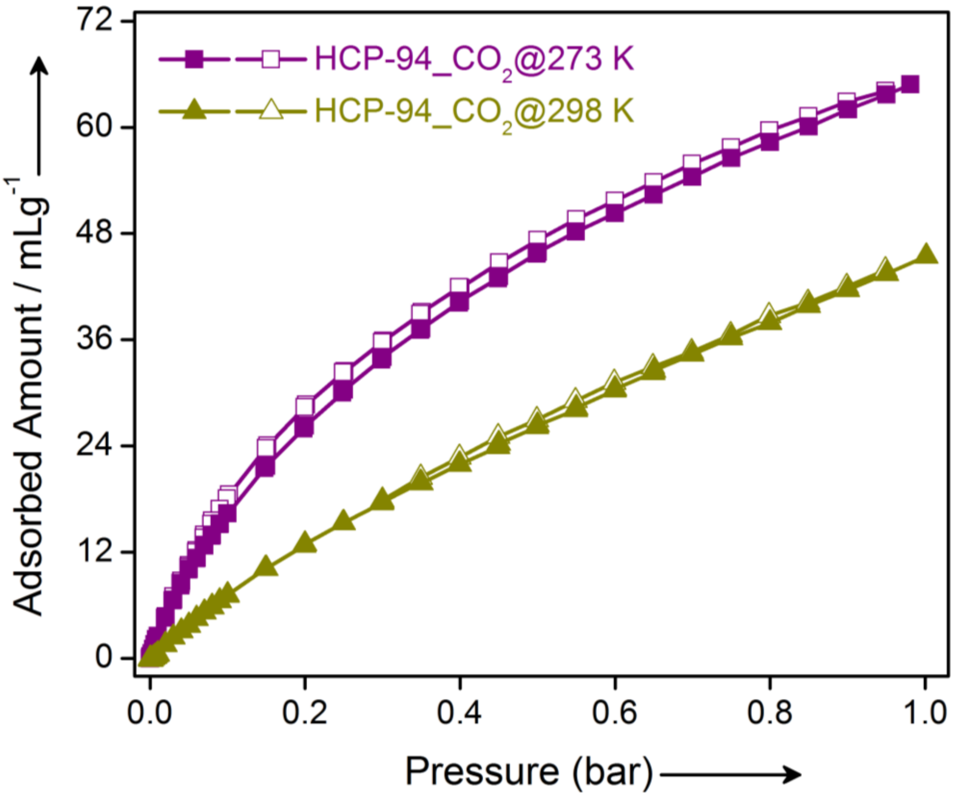

Figure S10: CO_2 adsorption isotherms at 273 K (purple) and 298 K (dark yellow) for HCP-94.

Table S1

Compounds	S_{BET} $m^2\ g^{-1}$	CO_2 at 273 K $mL\ g^{-1}$	CO_2 at 298 K $mL\ g^{-1}$	*Qst* $kJ\ mol^{-1}$
HCP-91	1028	74	43	30.7
HCP-94	672	65	45	32
